# Crop production kept stable and sustainable with the decrease of nitrogen rate in North China Plain: An economic and environmental assessment over 8 years

**DOI:** 10.1038/s41598-019-55913-1

**Published:** 2019-12-18

**Authors:** Zheng Liu, Ningning Yu, James J. Camberato, Jia Gao, Peng Liu, Bin Zhao, Jiwang Zhang

**Affiliations:** 1 State Key Laboratory of Crop Biology and College of Agronomy, Shandong Agricultural University, Tai-an, Shandong 271018 PR China; 2 Agronomy Department, Purdue University, 915 W State Street, West Lafayette, IN 47907 USA

**Keywords:** Environmental sciences, Solid Earth sciences

## Abstract

In pursuit of maximum grain yield farmers in the North China Plain usually apply excessive N fertilizer, resulting in wasted resources and environmental pollution. To assess the economic and environmental performances of different nitrogen rates will be conductive to sustain cleaner crop production. An 8-year field experiment was carried out with four treatments, N0 (0 kg ha^−1^ for winter wheat and summer maize), N1 (168 kg ha^−1^ for winter wheat and 129 kg ha^−1^ for summer maize), N2 (240 kg ha^−1^ for winter wheat and 185 kg ha^−1^ for summer maize) and N3 (300 kg ha^−1^ for winter wheat and summer maize), on the double cropping at Dawenkou research field (36°11’N, 117°06’E), Shandong Province, China. The crop production, soil physical-chemical parameters, and greenhouse gas emission are measured and the economic and environmental performances are assessed. The optimal nitrogen rate obtained the highest grain yield of summer maize in 4 of 8 year and was equivalent to conventional N rate in the other years. The nitrogen partial factor productivity and agronomic efficiency of optimal nitrogen rate was 63% and 58% higher than that of conventional nitrogen rate. The optimal nitrogen rate effectively decreased soil bulk density and increased weight percentage of water-stable aggregate and activities of urease and invertase compared to conventional nitrogen rate, which improved soil productivity. The fertilizer nitrogen loss and global warming potential of optimal nitrogen rate reduced by 76% and 35% compared to conventional nitrogen rate. The annual greenhouse gas intensity of optimal nitrogen rate decreased by 14–35% compared to others. The net ecosystem economic budget under optimal nitrogen rate is 252–604 $ ha^−1^ yr.^−1^ higher than other addition levels. The optimal nitrogen rate produces more grains and obtains higher economic and environmental benefits.

## Introduction

In the 21st century, more than half of the crop yield increase worldwide arises from the application of chemical fertilizers^1^. Nitrogen (N) is the nutrient that has the largest and most frequent beneficial effects on grain yield of summer maize^2^. In the North China Plain (NCP), farmers typically apply 250 to 350 kg N ha^−1^ for summer maize and 300 kg N ha^−1^ for winter wheat, 60% more than the recommended N rate^3^. At these rates, N removal from the field is only 16 to 40% of that added^4^. Nitrogen losses and greenhouse gas emission from farmland result in resource waste and air and water pollution^5^. The conventional application rate of N fertilizer results in high amounts of soil NO_3_-N at the end of the season, which can easily leach below the rootzone with rainfall and irrigation, and/or enter the atmosphere through ammonia volatilization^6^, or nitrification-denitrification resulting in atmospheric pollution^7^. Therefore, improving the utilization efficiency of N fertilizer is important for national food security and environmental quality^8^.

Fertilizers not only promote growth and development of crop but also alter soil productivity and greenhouse gas emission^9^. Some ions, such Cl^−^, CO_3_^2−^, and Ca^2+^ from fertilizers will influence compounds in soil associated with bulk density, aggregation, and so on^10,11^. In addition, the soil bulk density^12,13^, concentration of NO_3_-N and total N^14^, organic carbon^15^, C/N ratio^7,16^ and microbial activity^17^ also affected emission of CO_2_, CH_4_ and N_2_O. The greenhouse effect of greenhouse gas emission seriously threatens the future agricultural development and food security. There are many studies about effects of soil properties on crop growth and production. The high soil bulk density significantly decreases soil nutrient availability^18^. A bulk density of 1.5 g cm^−3^ in a silt loam soil restricts maize root growth^19^. The growth and development of roots can decrease bulk density^20^. The moderate bulk density and more aggregates build water and nutrient preserving capacity^21^. Soil enzymes are sensitive to environmental changes, which are driving force of nutrient conversion and are effective nutrient reserve^22^. Urease catalyze hydrolysis of urea and its activity reflects soil N status. Invertase activity reflects the accumulation and decomposition of soil organic carbon^23,24^. Higher activities of soil enzymes accelerate energy conversion and material cycling. The previous researched reported effects of N application amount on maize growth and differences in soil characteristic between unfertilized and fertilized farmland^25^.

Previous studies^3,26,27^ demonstrated the average optimal N rate for grain yield was 185 kg ha^−1^ for summer maize and 240 kg ha^−1^ for winter wheat. The hypothesis is that optimal N rate improves soil properties, decreases greenhouse gas emission and increases net ecosystem economic budget in the maize-wheat cropping system with straw returning relative to local traditional pattern.

## Materials and Methods

### Experiment site

**Figure 1:**
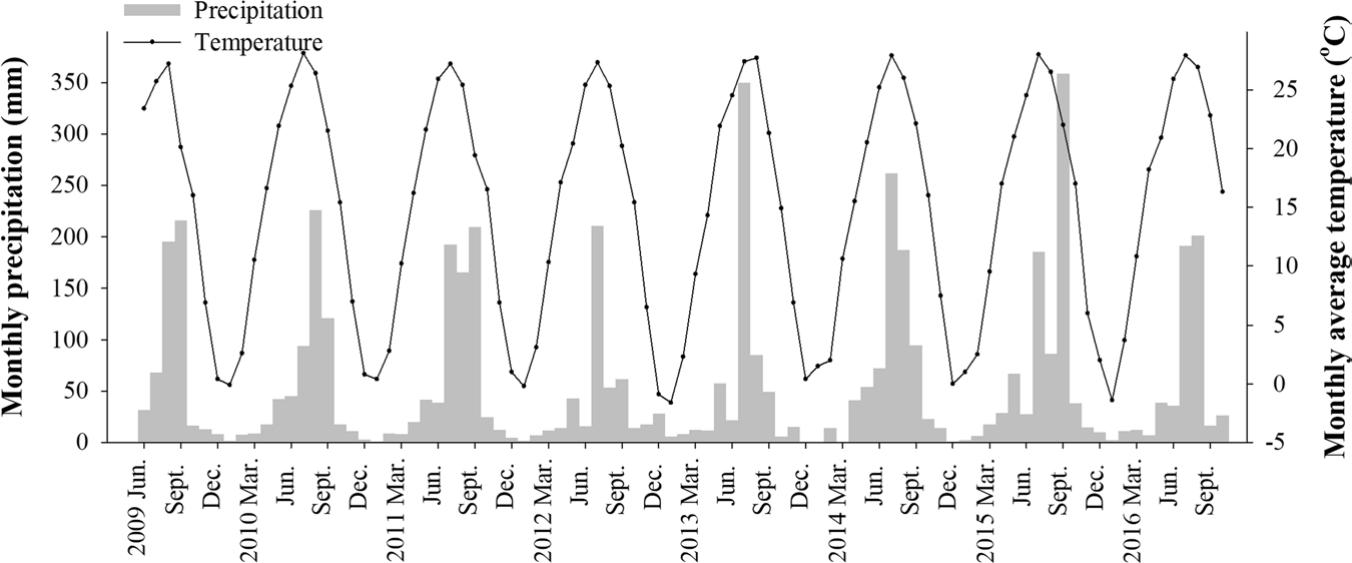
Monthly precipitation and average temperature in 2009–2016.

A field experiment was conducted from 2009 through 2016 on a brown loam soil (Typic Paleustalfs) in Dawenkou, China (36°11′N, 117°06′E, 178 m elevation) to determine the effects of N rate on crop productivity, soil properties and greenhouse gas emissions. This region has a temperate continental monsoon climate. Monthly precipitation and average temperature in 2009–2016 are shown in Fig. 1 and Supplement Table 1.

### Treatment application and field management

Prior to establishing the experiment, the field was in a wheat-maize rotation for 23 years. Both maize and wheat received 200 kg N ha^−1^ and 100 kg P ha^−1^, approximately. In the double-cropping of winter wheat-summer maize, straw from the previous crop was incorporated with rotary tillage prior to planting every season (straw amount in Supplement Fig. 1). Maize ‘Zhengdan958’ was planted at 7.5 × 10^4^ seeds ha^−1^ (i.e. 25 kg seed ha^−1^). Seeding of maize was approximately June 15 and harvest was in the month of October in 2009 through 2016 seasons. Plots were 10 60-cm rows of maize wide by 40 m long. During summer maize season, the nitrogen rates were 0 (N0), 129 (N1), 185 (N2), and 300 (N3) kg N ha^−1^, respectively. Of the total N rate, application timing was approximately 15% before seeding, 50% at the six leaf-stage (V6), and 35% at tasseling (VT), as shown in Table 1. Beginning in 2012, the fixed one quarter of each N0 plot (6 m wide by 10 m long) received the N2 treatment rate and is designated RN2. The conventional N rate for summer maize of smallholder farmers in this region was about 300 kg ha^−1^ ^3^. The recommended N rate established in previous studies was 185 kg ha^−1^ ^3^. Additions of phosphorus (P) and potassium (K) were 30 kg ha^−1^ before seeding, and 26 kg ha^−1^ and 71 kg ha^−1^, respectively at V6. Another 30 kg K ha^−1^ was applied at VT.

**Table 1:** Timing and rate of nitrogen application (kg ha^−1^) for summer maize and winter wheat.

Crop	Treatment	Timing and rate of nitrogen application (kg ha^−1^)			
		Before seeding	Jointing	Tasseling (maize)	Total
Winter wheat	N0	0	0	—	0
	N1	50	118	—	168
	N2 & RN2^†^	72	168	—	240
	N3	90	210	—	300
Summer maize	N0	0	0	0	0
	N1	21	63	45	129
	N2 & RN2^†^	30	90	65	185
	N3	50	145	105	300

^†^Nitrogen rates in the RN2 treatment were 0 kg N ha^−1^ in 2009–2011 and were 240 and 185 kg N ha^−1^ in 2012–2016 for wheat and maize, respectively.

Winter wheat (‘Tainong18’) seeded at 450 × 10^4^ seeds ha^−1^ in 25-cm wide rows on October 12 and harvested on June 12. Nitrogen fertilization rates were 0, 168, 240, and 300 kg N ha^−1^, 30% before seeding and the remainder at joint stage. Total P and K applied to each treatment were 150 kg P ha^−1^ and 120 kg K ha^−1^. The P was applied before seeding. The K application was split-applied, 72 kg K ha^−1^ before planting and 48 kg K ha^−1^ at joint stage.

All fertilizer was applied banded between the rows or corn, 5 cm deep. Fertilizers were urea, calcium superphosphate and potassium chloride. During wheat growing season, three irrigations occurred at sowing, overwintering and jointing, and each irrigation was 75 mm. The irrigation was 80 mm after maize seeding. There were no obvious diseases, pests and weeds during the experiment.

### Sampling and measurements

#### Crop production

Twenty wheat plants were collected from each plot center at maturity stage in 2016 and 5 maize plants were collected at VT and physiological maturity stage (R6) in 2009–2016. Grain yield (GY, 14% moisture content) and dry matter weight were measured as described by Liu *et al*.^26^.

$${\rm{GY}}={\rm{Ear}}\,{\rm{number}}\,{\rm{per}}\,{\rm{hectare}}\times {\rm{Kernel}}\,{\rm{number}}\,{\rm{per}}\,{\rm{ear}}\times {\rm{Thousand}}\,{\rm{kernel}}\,{\rm{weight}}$$

The total N concentration of plant samples was determined by the Kjeldahl method^28^. The contribution proportion (NCP) and efficiency (NTE) of nitrogen transport and nitrogen assimilation and after anthesis (NA) were calculated in the manner of Liu *et al*.^29^ and Shi *et al*.^30^. Nitrogen partial factor productivity (PFP_N_) and nitrogen agronomic efficiency (AE_N_) were calculated as following equation:

$${{\rm{PFP}}}_{{\rm{N}}}=\frac{GY}{NR}$$

$${{\rm{AE}}}_{{\rm{N}}}=\frac{GY-G{Y}_{0}}{NR}$$

where *GY* was grain yield with N fertilizer (kg ha^−1^), *GY*_0_ was grain yield without N fertilizer (kg ha^−1^), and *NR* was N fertilizer rate (kg ha^−1^).

#### Soil physical-chemical properties

Soil physical properties, bulk density and water-stable aggregates were measured after harvesting summer maize in 2015 and 2016. The samples from undisturbed soil for 0–30, 30–60, and 60–90 cm layers were collected using a steel cylinder of 100 cm^3^ volume (5 cm in diameter, and 5.1 cm in height) to determine bulk density^31^. Soil samples were taken in 0–30, 30–60 and 60–90 cm layers, respectively, using a metal cylinder with diameter of 25 cm, from three randomly selected spots each plot. The aggregates included particles in diameter from 0.25 to 10 mm, which was estimated using the wet-sieving technique as described by Haynes^32^. The fractal feature of water-stable aggregates was estimated by following formula according to Perfect *et al*.^33^:

$${\rm{D}}=3-\frac{{\rm{lg}}({m}_{i}/{m}_{0})}{{\rm{lg}}({\bar{d}}_{i}/{\bar{d}}_{max})}$$

where D was the fractal dimension of water-stable aggregate, m*i* is the cumulative weight of aggregates larger than ${\bar{d}}_{i}$ in diameter, m_0_ is the cumulative weight of all aggregates, ${\bar{d}}_{i}$ is the average of the adjacent aggregate diameters, and ${\bar{d}}_{max}$ is the average of the largest aggregate diameters.

Soil organic carbon and nutrients were measured after harvesting summer maize in 2009–2016. Five random soil cores (5 cm diameter) were taken in each plot from 0–30, 30–60, and 60–90 cm soil layers. Cores were pushed through a 2 mm sieve, air-dried, and then ground to pass a 0.25 mm sieve. Chemical analyses were conducted in triplicate and are expressed on an oven-dry basis. Soil organic carbon concentration was measured using dichromate oxidation method^34^, and soil organic matter concentration was equal to organic carbon concentration times Van Bemmelen factor, i.e. 1.724. Soil total N concentration was measured using the Kjeldahl digestion procedure^35^. Total K and available K were determined by atomic absorption spectrophotometry^36^. Total P was determined by the molybdenum blue colorimetric method following HClO_4_ digestion^37^. Available P was determined by the same method after extraction with 0.5 M NaHCO_3_^38^. 6 g fresh soil was extracted with 50 mL 1 mol L^−1^ KCl solution, then NO_3_^−^ is measured by the method of Alberts *et al*.^39^. Urease and invertase activities were measured as described by Tabatabai and Bremner^40^.

The fertilizer N loss (FNL, kg N ha^−1^) were calculated according to Liu *et al*.^26^.

$$\begin{array}{rcl}{\rm{FNL}} & = & Nitrogen\,loss\,{\rm{with}}\,{\rm{N}}-Nitrogen\,loss\,{\rm{without}}\,{\rm{N}}\\  & = & N\,rate-(Grain\,N\,content\,{\rm{with}}\,{\rm{N}}-Grain\,N\,content\,{\rm{without}}\,{\rm{N}})\\  &  & -(SN\,{\rm{with}}\,{\rm{N}}-SN\,{\rm{without}}\,{\rm{N}})\end{array}$$

Briefly, N content in 0–90 cm soil layer (SN, kg N ha^−1^) was calculated by soil bulk density, layer thickness and soil total N concentration. FNL was the difference in N losses between treatments receiving N and N0. Nitrogen losses were N application rate minus soil N surplus and grain N content, and soil N surplus was the interannual change in SN. The detailed formula derivation referred to Liu *et al*.^26^.

#### Greenhouse gas emission, global warming potential and greenhouse gas intensity

CO_2_, CH_4_ and N_2_O flux rates were determined using opaque plastic static chambers (50 × 50 × 40 cm) located between maize rows and a smaller chamber (50 × 20 × 40 cm) placed between wheat rows. Gas was collected daily at 9:00-9:30 am to avoid bias due to rising temperature during the morning hours during the week of fertilization in the 2015–2016. Additional collections were added after heavy precipitation. Each sampling was conducted in 10 min intervals for a total of 30 min^41^. The greenhouse gas concentrations were measured using a chromatograph meter (Shimadzu GC-14B, Japan). The greenhouse gas flux rates were calculated based on liner regression^41^, as following:

$${\rm{F}}=\frac{{\rm{d}}C}{{\rm{d}}t}\times \frac{mPV}{ART}=H\times \frac{{\rm{d}}C}{{\rm{d}}t}\times \frac{mP}{RT}$$

where F was the flux rate (mg m^−2^ h^−1^ for CO_2_ and CH_4_ or μg m^−2^ h^−1^ for N_2_O), and $\frac{{\rm{d}}C}{{\rm{d}}t}$ was the ratio of greenhouse gas concentration to sampling time (ppm h^−1^ for CO_2_ and CH_4_ or ppb h^−1^ for N_2_O). Parameters *H*, *m*, *P*, *R*, *T* were chamber height (m), molecular weight (g mol^−1^), atmospheric pressure (Pa), gas constant (J mol^−1^ k^−1^) and temperature (K).

The cumulative greenhouse gas emissions (g m^−2^ for CO_2_ and CH_4_ and mg m^−2^ for N_2_O) were calculated by linearly interpolating the flux rate to collection dates.

The atmospheric carbon sequestration (ACS), global warming potential (GWP) and greenhouse gas intensity (GHGI) were calculated^41–43^.

#### Net ecosystem economic budget

The net ecosystem economic budget was calculated^44^:

$${\rm{NEEB}}=Net\,return\,to\,N-N\,Fertilizer\,costs-GWP\,costs$$

The price of urea, maize grain and wheat grain were 0.58 $ kg^−1^ N, 0.29 $ kg^−1^ and 0.29 $ kg^−1^, respectively^45^. *Net return to N* was the increase in yield with N multiplied by the price of grain. *GWP costs* were calculated by carbon-trade prices, 17 $ t^−1^ CO_2_-eqv^44^ and GWP. To simplify, this study focused on the differences in NEEB between nitrogen rates (N1, N2 and N3) and the N0 treatment.

### Statistical analysis

Data were analyzed by analysis of variance (ANOVA) procedure using SPSS 17.0 software (SPSS Inc., Chicago, IL, USA) with P ≤ 0.05 considered significant. Treatments were compared with Duncan’s multiple range test (P ≤ 0.05).

## Results

### Crop Production

**Figure 2:**
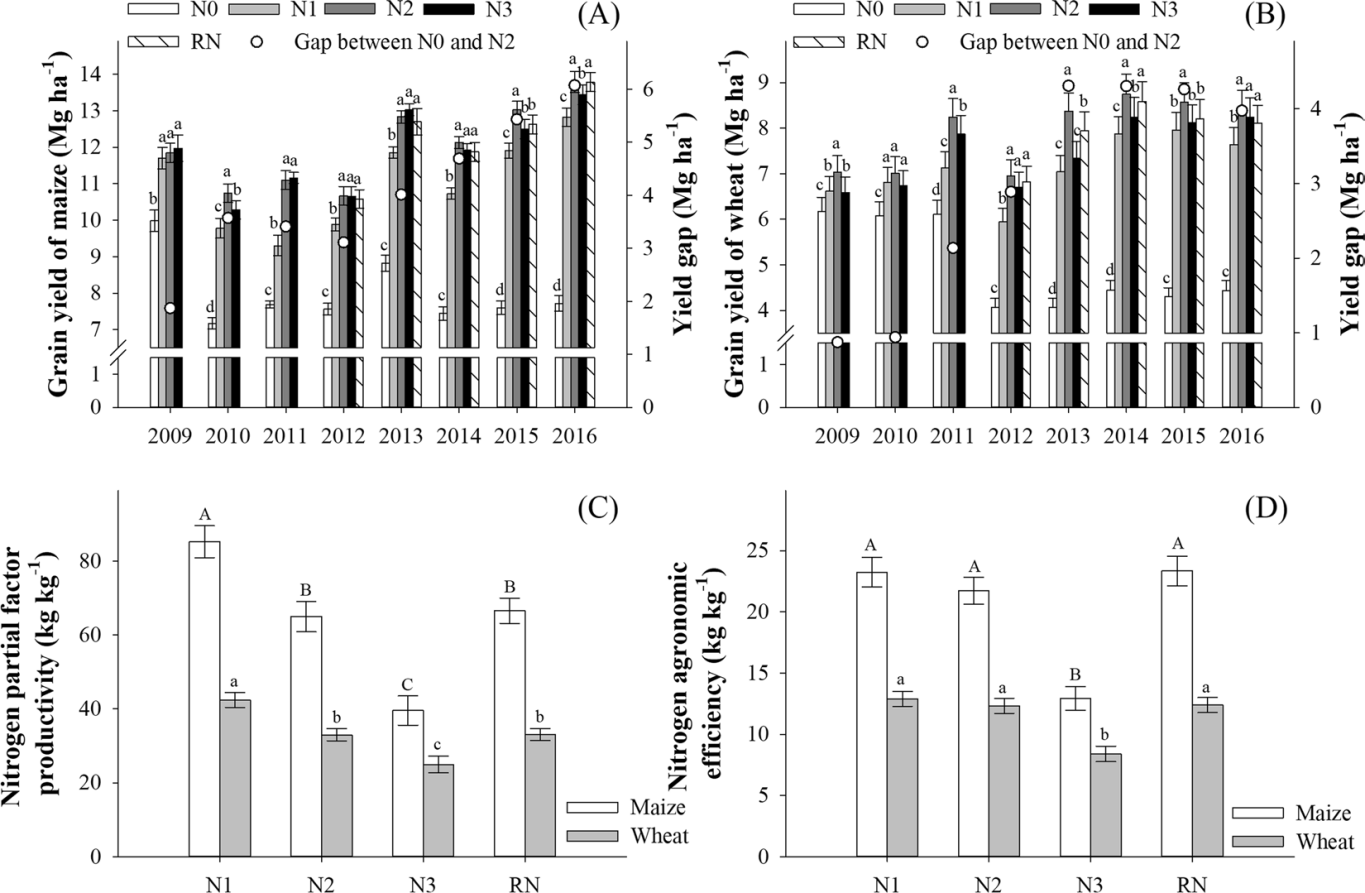
Grain yield of summer maize (**A**) and winter wheat (**B**), nitrogen partial factor productivity (**C**) and nitrogen agronomic efficiency (**D**) as affected by nitrogen rate. Nitrogen rates in wheat and maize, respectively, are; 0 and 0 kg N ha^−1^ (N0), 168 and 129 N ha^−1^ (N1), 240 and 185 kg N ha^−1^ (N2), and 300 and 300 kg N ha^−1^ (N3) in 2009–2016. Nitrogen rates under RN2 treatment in wheat and maize are 240 and 185 kg N ha^−1^ in 2012–2016. Bars with different capital or small letters are significantly different at the p ≤ 0.05 level. Error bars denote the standard deviation. Results in (**C**,**D**) are the average over 8 years.

Grain yield under N0 for summer maize varied substantially from 7.2 to 10.0 Mg ha^−1^ across all years, but in 6 of 8 years yield ranged narrowly between 7.2 and 7.7 Mg ha^−1^, and that for winter wheat ranged from 6.1 to 6.2 Mg ha^−1^ in 2009–2011 and from 4.1 to 4.4 Mg ha^−1^ in 2012–2016 (Fig. 2A,B). The grain yield under N1 was decreased compared to N2 in all years except for maize in the first year and for wheat in the second year. The yield components (ears per hectare, kernels per ear and thousand-kernel weight) for maize increased with N rate, but N2 and N3 did not differ (Supplement Table 2). The yield gap between N0 and N2 for maize increased from 1.9 Mg ha^−1^ in 2009 to 6.1 Mg ha^−1^ in 2016. The yield gap between N0 and N2 for wheat was about 0.9 Mg ha^−1^ in 2009–2010 and 4.0–4.3 Mg ha^−1^ in 2013–2016, and increased linearly in 2010–2013. The grain yield under N3 for maize and wheat was less than or equal to that under N2. Moreover, N2 promoted accumulation and distribution of dry matter and nitrogen contributing to grain yield compared to other treatments (Supplement Fig. 2, Supplement Tables 3–5). The grain yield of RN for maize and wheat was not different from N2 in 3 of 5 years.

As shown in Fig. 2C, the average PFP_N_ for maize and wheat decreased with N rates. The average PFP_N_ under N2 for maize and wheat decreased 24% and 22%, respectively, compared to N1, and increased 64% and 32%, respectively, compared to N3. There was no significant difference in the average PFP_N_ between N2 and RN for maize and wheat. As for the average AEN, N3 obtained the least efficiency, i.e. 13 kg kg^−1^ for maize and 8 kg kg^−1^ for wheat. The other treatments did not differ in the average AE_N_ (Fig. 2D).

### Soil physical-chemical properties

**Figure 3:**
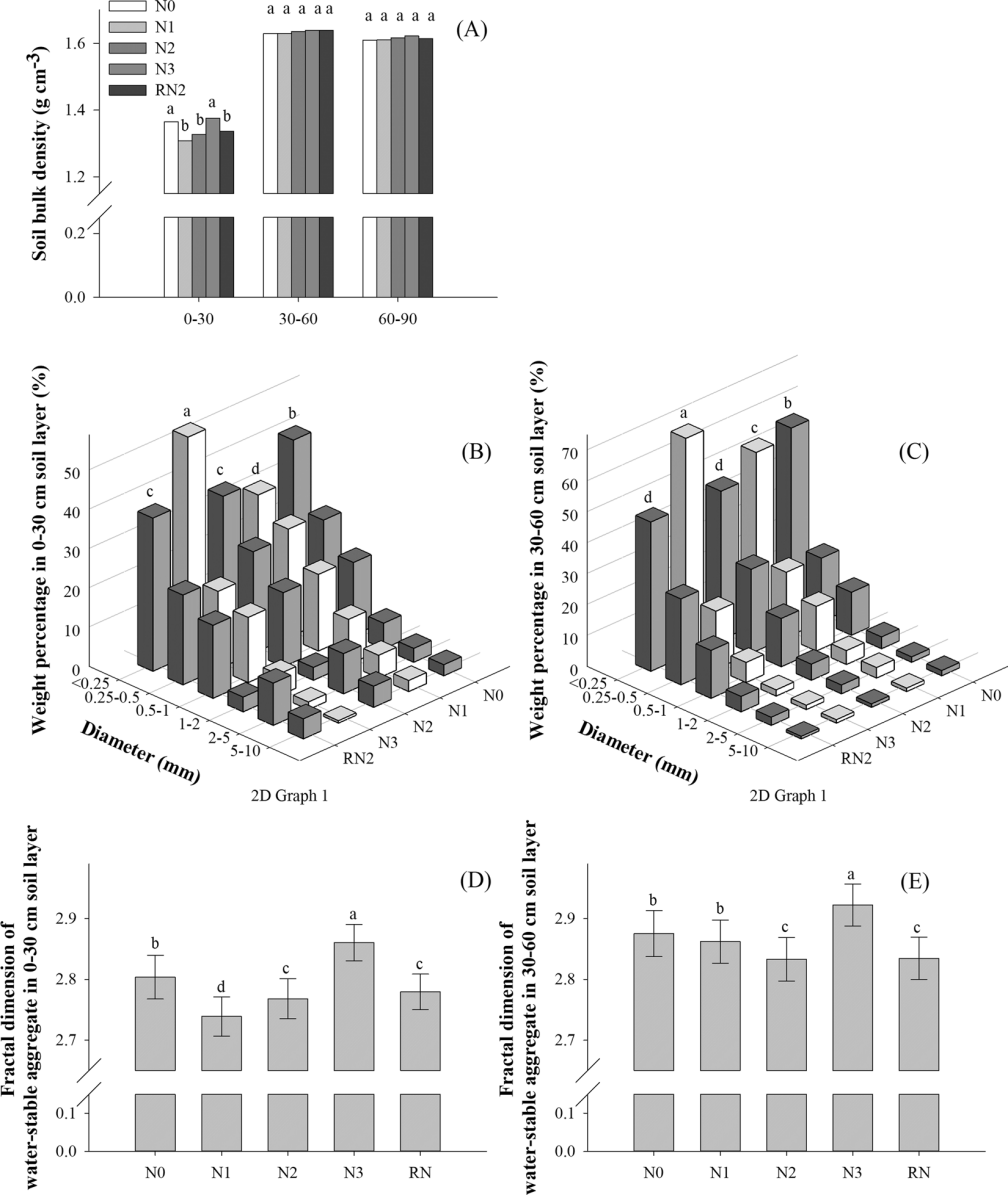
Soil bulk density (**A**), weight percentage of water-stable aggregates and fractal dimension at 0–30 cm depth (**B** and **D**), and at 30–60 cm depth (**C** and **E**) with different nitrogen rates. Nitrogen rates in wheat and maize, respectively, are; 0 and 0 kg N ha^−1^ (N0), 168 and 129 N ha^−1^ (N1), 240 and 185 kg N ha^−1^ (N2), and 300 and 300 kg N ha^−1^ (N3) in 2009–2016. Nitrogen rates under RN2 treatment in wheat and maize are 240 and 185 kg N ha^−1^ in 2012–2016. Bars with different capital or small letters are significantly different at the p ≤ 0.05 level. Error bars denote the standard deviation. Results in Fig. 2C,D are the average over 8 years.

Soil physical-chemical properties were measured after 8 years of different N rates. Nitrogen rate affected soil bulk density (BD) in the 0–30 cm soil layer, but not in the 30–60 or 60–90 cm soil layers (Fig. 3A). All N rates, except N3, decreased BD of the 0–30 cm depth approximately 0.03–0.06 g cm^−3^, compared to the N0. Soil BD under N0 and N3 did not differ, averaging about 1.38 g cm^−3^.

Nitrogen rate affected the weight percentage of water-stable aggregates (WSA) at 0–30 and 30–60 soil depths (Fig. 3B,C), but not at the 60–90 cm depth (data not shown). At 0–30 cm, WSA under N2 was 6% higher than that under N1 and 41% than that under N3 (Fig. 3B). At 30–60 cm, the effects of N treatments on WSA were similar to the top layer except N3 had lower WSA than N0 (Fig. 3C). In Fig. 3D,E, the fractal dimension of WSA (*D*) ranged among treatments from 2.74 to 2.86 at 0–30 cm depth and from 2.83 to 2.92 at 30–60 cm depth. In 0–30 cm soil layer, *D* at N2 decreased by 1.3% and 3.2% compared to N0 and N3, respectively, and increased by 1.1% related to N1. At 30–60 cm soil depth, *D* at N2 was the lowest in comparison with other treatments (Supplement Table 3).

**Figure 4:**
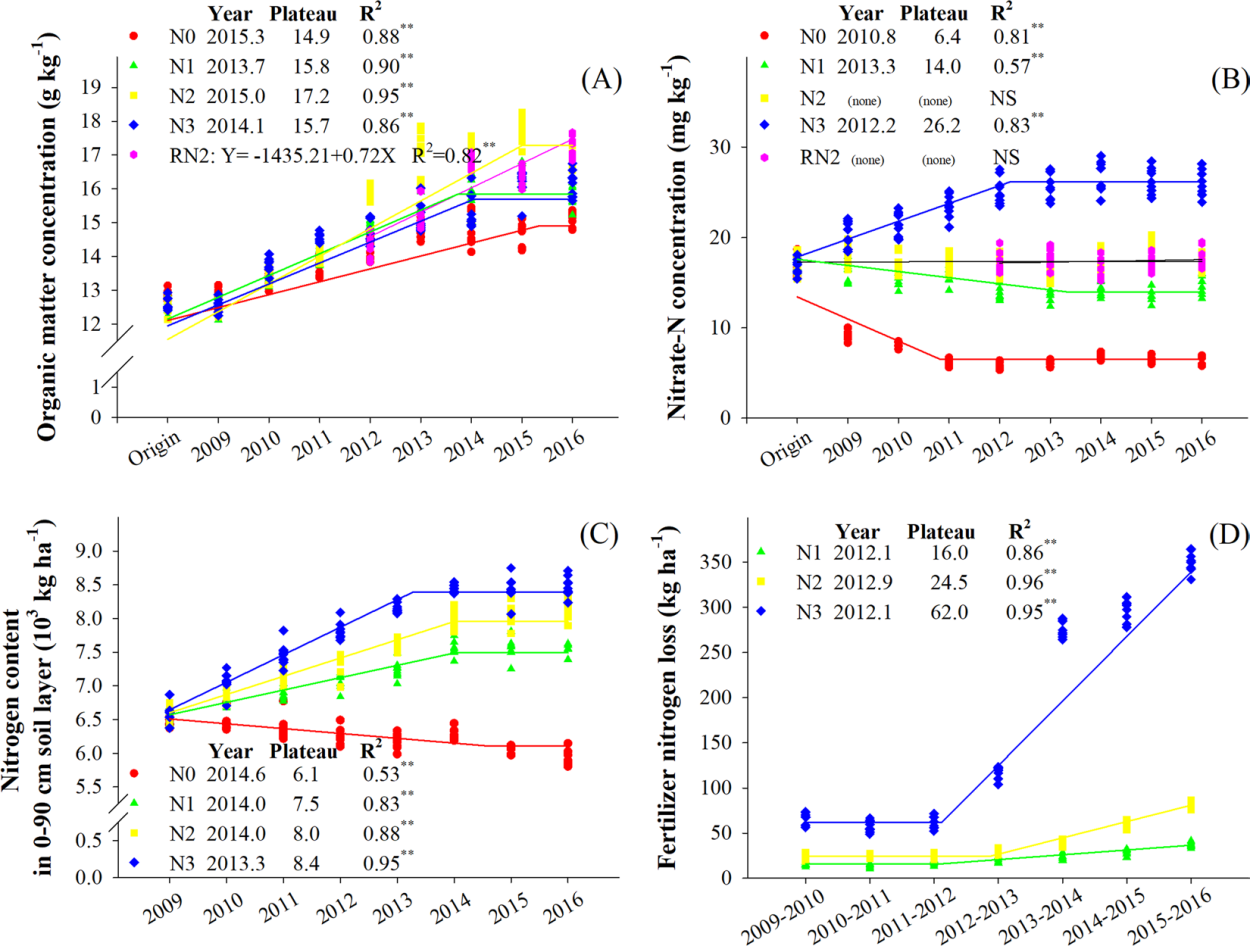
Effects of N rates on organic matter (**A**), nitrate-N concentration (**B**) in 0–30 cm soil layer, nitrogen content in 0–90 cm soil layer (**C**) fertilizer nitrogen loss and (**D**) at maize physiological maturity. Nitrogen rates in wheat and maize, respectively, are; 0 and 0 kg N ha^−1^ (N0), 168 and 129 N ha^−1^ (N1), 240 and 185 kg N ha^−1^ (N2), and 300 and 300 kg N ha^−1^ (N3) in 2009–2016. Nitrogen rates under RN2 treatment in wheat and maize are 0 and 0 kg N ha^−1^ in 2009–2011, and 240 and 185 kg N ha^−1^ in 2012–2016. Origin: June 2009. Each point is a plot mean. Year: the time to reach plateau value. Plateau: plateau value. NS: Not significant, P > 0.05. **Significant at the 0.01 probability level.

Organic matter concentration of all treatments increased with years and reached a plateau after 2014–2015 for all treatments but RN2 (Fig. 4A). Soil organic matter under N2 increased faster and N0 grew slowly. The maximum concentration under N0, N1, N2 and N3 treatments was 14.9, 15.8, 17.3 and 15.7 g kg^−1^, respectively. The organic matter concentration under RN2 increased with experimental years and reached 17.4 g kg^−1^ in 2016.

The nitrate-N concentration in the upper 30 cm of soil under N0 and N1 decreased over time while that under N3 significantly increased (Fig. 4B). Notably, the nitrate-N concentration under N2 and RN2 remained stable. After 8 years of experiment, total N content in 0–90 cm soil layer (SN) under N1, N2 and N3 increased by 17, 24, and 28%, and that under N0 decreased by 9%.

Fertilizer N loss (FNL) did not increase in the first 3-4 years of experiment. Thereafter, FNL increased over time (Fig. 4D, Supplement Table 6), subtly under N1 and N2, but substantially under N3. As for P and K in the 0–30 cm soil layer, the concentration of total P and available K increased and the concentration of total K and available P remained stable with years (Supplement Table 7). The activities of urease and invertase increased with increase in N rate, which promoted the recycling of C and N in the soil. However, N3 reduced their activity in the 0–30 cm soil depth (Supplement Table 8).

### Greenhouse gas emissions

**Figure 5:**
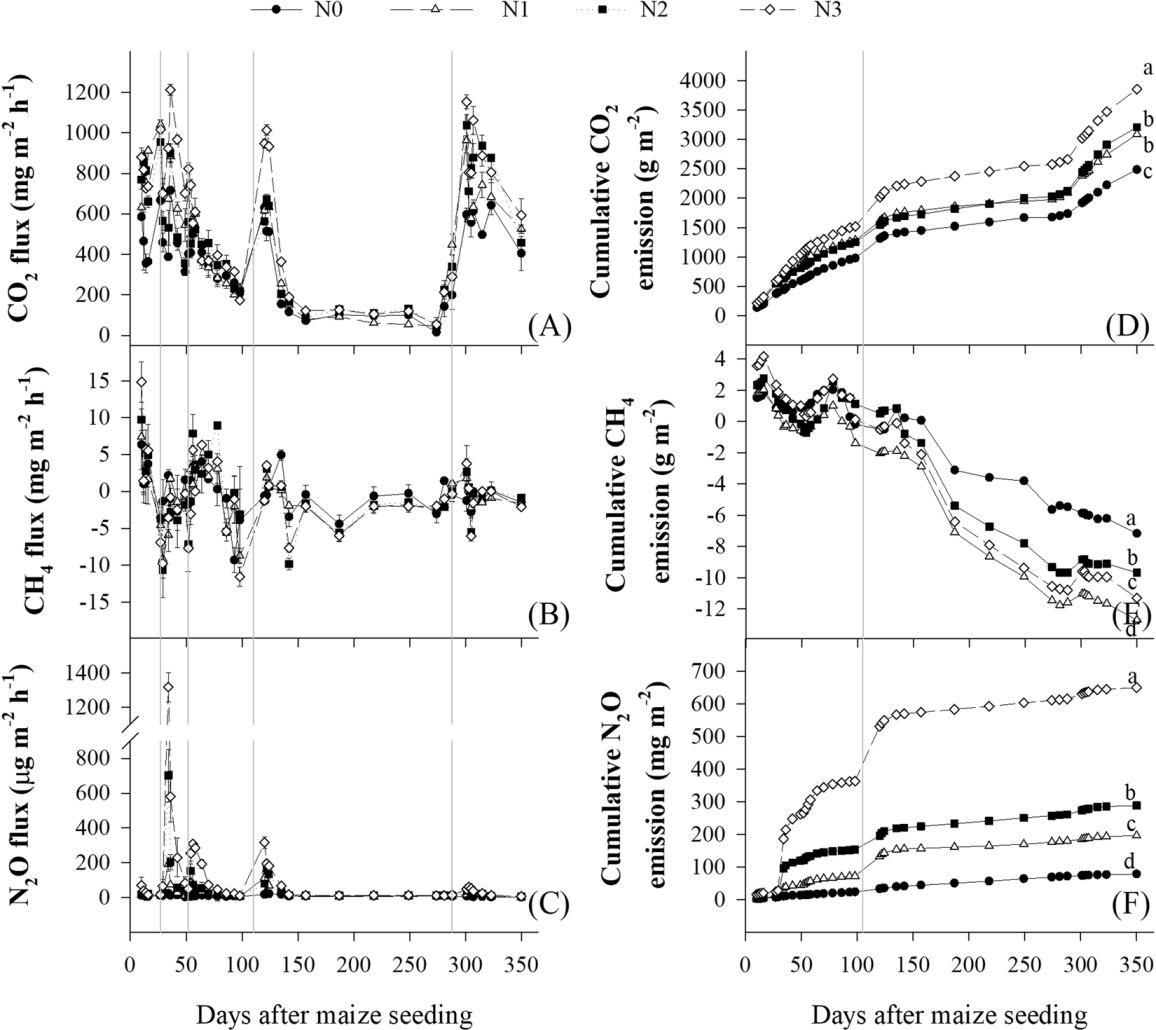
Flux rate and cumulative amount of CO_2_ (**A**,**D**), CH_4_ (**B**,**E**) and N_2_O (**C**,**F**) under different nitrogen rates during 2015–2016 summer maize and winter wheat growing seasons. Nitrogen rate in wheat and maize, respectively, were; 0 and 0 kg N ha^−1^ (N0), 168 and 129 N ha^−1^ (N1), 240 and 185 kg N ha^−1^ (N2 and RN), and 300 and 300 kg N ha^−1^ (N3). Error bars denote the standard deviation. In (**A**–**C**) the vertical gray lines represent fertilizer application timings. In (**D**–**F**) the left side of the vertical gray line represents summer maize season, and the right side represents winter wheat season. Treatments with different lower-case letters are significantly different at the p ≤ 0.05 level.

Nitrogen rate significantly influenced CO_2_, CH_4_ and N_2_O emission flux (Fig. 5A–C). The CO_2_ flux under N0 was lowest and that under N3 was largest in wheat and maize growing period. The CO_2_ flux of all treatments (including N0) increased rapidly after each application of fertilizer. Cumulative CO_2_ emissions increased with N rate (Fig. 5D). CH_4_ flux rates were both positive and negative (Fig. 5B). After the earliest measurements cumulative CH_4_ declined for many measurements before increasing again. In the summer maize season, CH_4_ flux rates under N2 and N3 were higher compared to N0 and N1. In winter wheat season, CH_4_ flux rates under N0 and N2 were more often higher than that under N1 and N2. Cumulative CH_4_ absorption (negative values of emission) decreased in order N1 > N3 > N2 > N0. N_2_O flux of N1, N2 and N3 treatments increased rapidly after fertilizing, with higher N application rates inducing higher rates of N_2_O flux (Fig. 5C). There was no significant increase in N_2_O flux coincident with only P and K fertilization under N0. The cumulative N_2_O emission increased with N rate (Fig. 5F). N1 and N2 increased N_2_O emissions 2.5- and 3.7-fold compared to N0, while N3 increased N_2_O emissions 8.3-fold.

In the Table 2, global warming potential (GWP) for maize growing season under N2 increased by 20% compared to N0 and decreased by 28% related to N3, and that for wheat growing season under N2 was 42% less than that under N3. The annual GWP under N2 increased by 6% compared to N0 and decreased by 35% compared to N3. N1 and N2 did not differ in GWP regardless of periods. The greenhouse gas intensity (GHGI) under N2 for maize decreased by 20%, 11% and 29%, and that for wheat decreased by 35%, 19% and 40% compared to N0, N1 and N3, respectively. The annual GHGI under N2 decreased by 28%, 14% and 35% related to N0, N1 and N3, respectively.

**Table 2:** Global warming potential (GWP) and greenhouse gas intensity (GHGI) under different nitrogen rates for 2015–2016 summer maize and winter wheat growing seasons.

Treatment	GWP (10^3^ kg CO_2_-eqv ha^−1^)			GHGI (kg CO_2_-eqv kg^−1^)		
	Maize	Wheat	Annual	Maize	Wheat	Annual
N0	8.5 c	9.9 b	18.4 b	1.06 b	1.79 b	1.36 b
N1	10.5 b	9.7 b	20.2 b	0.96 c	1.42 c	1.14 c
N2	10.2 b	9.3 b	19.5 b	0.85 d	1.16 d	0.98 d
N3	14.2 a	15.9 a	30.1 a	1.20 a	1.93 a	1.50 a

Nitrogen rates in wheat and maize, respectively, are; 0 and 0 kg N ha^−1^ (N0), 168 and 129 N ha^−1^ (N1), 240 and 185 kg N ha^−1^ (N2), and 300 and 300 kg N ha^−1^ (N3) in 2009–2016. Values followed by a different small letter within column are significantly different at P ≤ 0.05.

### Net ecosystem economic budget

In comparison with N3, N2 improved the net ecosystem economic budget (Table 3). The net return to N under N2 and N3 did not differ, which were 667 and 696 $ ha^−1^ yr^−1^ more compared to N1. The GWP cost under N1 and N2 did not differ, and was 168 and 180 $ ha^−1^ yr^−1^ less than that under N3. The NEEB under N2 increased 1015, 604 and 252 $ ha^−1^ yr^−1^, respectively, compared to N0, N1 and N3.

**Table 3:** Net return to N, N fertilizer costs, global warming potential (GWP) costs and net ecosystem economic budget (NEEB) of N1, N2 and N3 treatments related to N0 treatment.

Treatment	Net return to N	N fertilizer costs	GWP costs	NEEB
	------------------------------------------------------ $ ha^−1^ yr^−1^ ------------------------------------------------------			
N1	1218 b	172	31 b	1015 c
N2	1885 a	247	19 b	1619 a
N3	1914 a	348	199 a	1367 b

Nitrogen rates in wheat and maize, respectively, are; 0 and 0 kg N ha^−1^ (N0), 168 and 129 N ha^−1^ (N1), 240 and 185 kg N ha^−1^ (N2), and 300 and 300 kg N ha^−1^ (N3) in 2009–2016. The results represent the differences between N treatments (N1, N2 and N3) and N0. The price of urea, maize grain and wheat grain are 0.58 $ kg^−1^ N, 0.29 $ kg^−1^ and 0.29 $ kg^−1^, respectively. Net return to N is the increase in grain yield with N multiplied by the price of grain. N fertilizer costs is the nitrogen rate multiplied by the price of urea. GWP costs are calculated based on carbon-trade prices, 17 $ t^−1^ CO_2_-equivalent. NEEB = Net return to N - fertilizer costs – GWP costs. Values followed by a different small letter within column are significantly different at P ≤ 0.05.

## Discussion

The farmers in the North China Plain apply fertilizers lavishly to get maximum crops yields, but lag behind in practices that increase efficiency of N fertilization^27^. Generally, only 5–15% of fertilizers are transformed into grain under high N application rates^46^. The remaining N is lost as gaseous emissions, or leached from the soil^47^, or immobilized by microorganisms^48^. Defining an N rate that optimizes profit not only benefits the farmer directly, but the whole of society environmentally.

In our study, dry matter, N translocation, partial factor productivity and agronomy efficiency under the optimal N rate (240 kg N ha^−1^ for wheat and 185 kg N ha^−1^ for maize) were significantly higher compared to the conventional N rate (300 kg N ha^−1^ to both maize and wheat), which is consistent with previous studies^27,49^. The conventional N rate decreased harvest index significantly, which likely led to reduced grain yield compared to the optimal N rate. According to previous research, the dry matter accumulation was the base of grain yield, and there was a positive correlation between grain yield and dry matter accumulation after anthesis, especially^50^. The optimal N rate improved dry matter weight, N accumulation and N translocation of summer maize, then increasing grain yield. The conventional N rate, compared to the optimal N rate, also decreased N harvest index and the contribution and efficiency of N translocation to the grain, while straw N content was increased. As a result, N partial factor productivity and N agronomic efficiency were lower under the conventional N rate relative to the optimal N rate. In summary, the N rate, 180 kg N ha^−1^ for summer maize and 240 kg N ha^−1^ for winter wheat, produced equal or more grain with less fertilizer cost compared to the conventional N rate. In addition, 70% of the optimal N rate decreased grain yield significantly.

Nitrogen rates not only influenced maize and wheat production by satisfying the nutritional requirement of the crop, but also altered soil physical-chemical properties. The optimal N rate resulted in higher soil organic matter under the condition of straw returning, compared to both lower and higher N rates. Increased soil organic matter likely resulted from larger root systems returning more residues to the soil^26^. The optimal N rate reduced soil bulk density and increased water-stable aggregation compared to the conventional N rate. Soil organic matter is important for bulk density and water-stable aggregates^51,52^. Bulk density affects soil water and air permeability affecting growth and development of the crop^20^. Soil organic matter concentration increases aggregation, especially water-stable aggregates^53,54^. Soil aggregation indirectly affect nutrient availability by adsorption, aeration and water retention^55^.

In addition, the optimal N rate obtained higher activities of urease and invertase in the 0–30 cm depth likely resulting from larger and more active root system^26^. However, their activities in 30–60 cm depth without N were the highest compared to other N rates. We cannot explain this phenomenon now but will pay attention to it in the future. Urease and invertase take part in soil N and C cycle and energy flow^56,57^, then promote crop production.

The N rate amount had slight effects on soil nutrient concentration (e.g. total P). Interestingly, the total and available K concentrations under optimal N rate were lower than that under other N rates, and also decreased significantly compared to levels in 2009. We suspect, on the one hand, much grain production consumes more K in the soil^58^, and potash is not enough to supplement consumption, which leads to a decrease in total and available K in the soil together; on the other hand, N affected cation exchange capacity by decreasing soil pH and pH-dependent charge increasing K leaching^59^. In comparison with the conventional N rate, the optimal N rate decreased nitrate-N concentration in 0–30 cm soil layer. Note that grain yield without N fertilizer decreased while soil organic carbon concentration increased and total N and NO_3_-N content decreased during the experimental years, which might indicate that the soil N content, not soil organic matter, was the limiting factor of grain yield in the absence of N fertilizer.

Nitrogen balance of crop-soil system reflect N inputs (e.g. seeds, irrigation, rainfall, wet deposition, biological nitrogen fixation and fertilization) and outputs (grain, runoff, leaching, ammonia volatilization and denitrification), which affects soil N pool and fertilizer N loss^26^. The N losses include runoff, leaching, ammonia volatilization and denitrification^27^. The fertilizer N loss defines as the difference in N losses between N rates and zero N rate. The fertilizer N fates include contribution to grain N, contribution to SN and fertilizer N loss (Supplement Table 6). The fertilizer N loss of all treatments increased with years resulting from the soil N pool was near saturation. The grain nitrogen content was relatively stable compared to the other two pathways. The increment in soil N content tapered off, especially under N3 treatment, that is, a saturated value might soon appear under the conventional N rate. So, the fertilizer N loss under all treatments increased gradually over time, and it under the optimal N rate was significantly less relative to conventional N rate. To sum up, the optimal N rate improved soil physical-chemical properties (e.g. bulk density, water-stable aggregate, and organic matter and nutrient concentration) which promoted soil productivity.

The N application rate increased CO_2_ (Fig. 5A,D) and N_2_O (Fig. 5C,F) emissions. Peak flux of both gasses tended to coincide with N application events (Fig. 5A,C). Cumulative emissions were increased from lowest to highest N rates by 55% and 725% for CO_2_ and N_2_O, respectively. Under 300 kg N ha^−1^ rate, the N_2_O emission factors defined as the percentage of N input convert to N_2_O emission are 1.4% and 0.8% in maize and wheat growing season, respectively. In this study, the CO_2_ emission arose from soil respiration only. Some studies reported that N fertilizer increased CO_2_ emissions^60^, while other studies showed that N fertilizer increased carbon sequestration and organic carbon concentration of soil^15^. This study has different findings. When N rates increased from 0, to 129 (deficient), to 185 kg ha^−1^ (optimal), the soil bulk density decreased and soil organic carbon increased, which increased CO_2_ emission. However, the conventional N rate (300 kg N ha^−1^) brought higher bulk density, less organic carbon and more CO_2_ emission compared to the optimal N rate, which was not our expectation. Why does more N rate continuously increased CO_2_ emission? At least it’s not because of bulk density and soil organic carbon. We guess that the higher N rate fueled eutrophic microorganism, and the latter produced more CO_2_. Because of the lack of direct evidence, this study cannot explain this phenomenon exactly.

Soil bulk density^13^, C/N ratio^7,16^ and microbial activity^17^ may have significant effects on greenhouse gas emissions. Briefly, bulk density affects the soil aeration, which choose aerobic and anaerobic microorganisms and determine redox state partly. Then, these changes promote or inhibit soil respiration, methane-producing/consuming organism activity, nitrification and denitrification, and other biological and chemical reactions. The soil nutrient state, pH and C/N ratio also play crucial roles during this process. The CH_4_-oxidizing bacteria absorb CH_4_ in the soil surface^61^. In comparison with N rates, growing seasons had more significant effects on CH_4_ emission (Fig. 5B,E). The CH_4_ flux rate increased significantly after straw incorporation. The cumulative CH_4_ emission was close to zero during the summer maize season, and that was negative during the winter wheat season, which was consistent with previous studies^62,63^. The NO_3_-N concentration^14^, C/N ratio^7^, anaerobic condition^12^ and denitrification potential in the 0–30 cm soil layer affect N_2_O emission. In this study, there was a close relationship between N rates and N_2_O emission (Fig. 5C,F). It was highly likely that N_2_O emissions arose from nitrification as well as denitrification. Urease catalyzed hydrolysis of N fertilizer (urea) to ammonium. Ammonium was oxidized by chemoautotrophic nitrifiers to produce nitrate, then nitrate was reduced by denitrifying microorganisms under anaerobic conditions. Both processes can generate N_2_O^64,65^.

The conventional N rate resulted in more NO_3_-N and total N in the 0–30 cm soil layer (Supplement Table 7). Meanwhile, the conventional N rate also led to higher bulk density and less water-stable aggregate, resulting in anaerobic conditions. The combination of above two increased N_2_O emission under conventional N rate. The concentration of NO_3_-N and total N decreased significantly with decrease in N rate, so the flux rate and cumulative emission also decreased significantly. The global warming potential and grain yield increased with N rates (Table 2). However, greenhouse gas intensity (global warming potential per grain yield) first decreased then increased with N rates. The optimal N rate obtained the less greenhouse gas intensity, that is, the optimal N rate produced more grain yield with less greenhouse gas. In comparison with conventional N rate, annual greenhouse gas intensity under optimal N rate decreased by 0.48 kg CO_2_-eqv per 1 kg grain yield.

**Figure 6:**
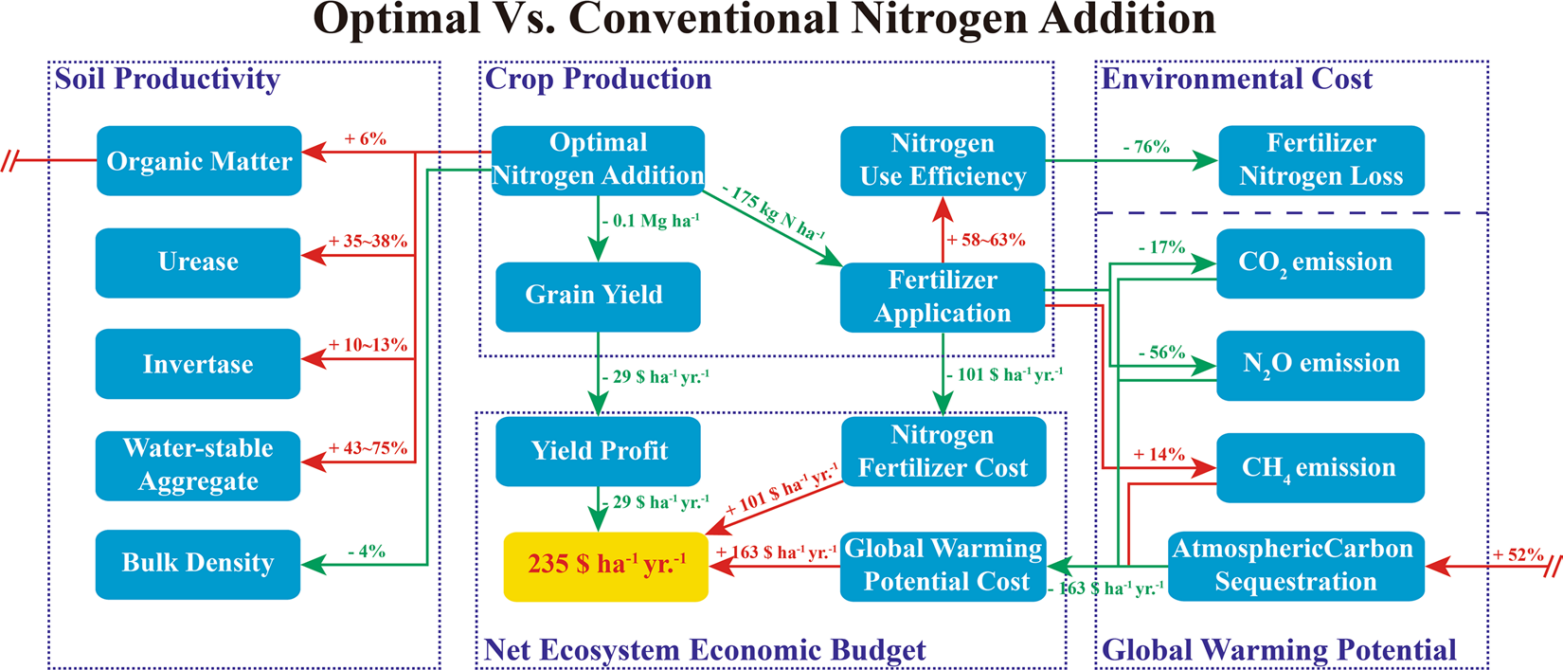
Optimal nitrogen addition improves soil productivity, decreases environmental costs, and increases net ecosystem economic budget compared to conventional nitrogen addition. Optimal nitrogen addition: 185 kg ha^−1^ for summer maize and 240 kg ha^−1^ for winter wheat; Conventional nitrogen addition: 300 kg ha^−1^ for summer maize and 300 kg ha^−1^ for winter wheat; The values near the arrow denote the effect on the variable in which the arrow points. The arrows reflect the reasons that promoted the changes of the variables. The red value on a yellow background indicates the difference in net ecosystem economic budget between optimal and conventional nitrogen addition.

Considering net return to N, N fertilizer costs and global warming potential costs, the net ecosystem economic budget was higher under the optimal N rate. The optimal N rate decreased costs of fertilizer and global warming potential, and increased net ecosystem economic budget compared to conventional N rate. Although the costs of fertilizer and global warming potential were the lowest under deficient N rate, its net return to N and net ecosystem economic budget were significantly lower relative to optimal N rate. To sum up, the optimal N rate produced more grain with less greenhouse gas, and obtained higher net ecosystem economic budget compared to other N rate levels (Fig. 6).

## Conclusion

This study comprehensively evaluates the environmental and economic benefits of nitrogen fertilizer application in the summer maize-winter wheat cropping system with straw returning. The optimal N rate, 185 kg ha^−1^ for summer maize and 240 kg ha^−1^ for winter wheat effectively improved soil physical-chemical properties for the past 8 years, and reduced fertilizer-induced nitrogen loss and greenhouse gas emission on the basis of ensuring crop production.

## Supplementary information

Supplement Information
