# Supplementary material for: Crop production kept stable and sustainable with the decrease of nitrogen rate in North China Plain: An economic and environmental assessment over 8 years
Source: Sci Rep. 2019 Dec 18;9:19335. doi: 10.1038/s41598-019-55913-1 (PMC6920463; doi:10.1038/s41598-019-55913-1)
Supplement: Supplementary file 1 — Supplement Information [file 41598_2019_55913_MOESM1_ESM.pdf]

Crop production kept stable and sustainable with the decrease of nitrogen rate in North China Plain: An economic and environmental assessment over 8 years

Zheng Liu[a, †], Ningning Yu[a, †], James J. Camberato[b], Jia Gao[a], Peng Liu[a], Bin Zhao[a], Jiwang Zhang[a,*]

[a]*State Key Laboratory of Crop Biology and College of Agronomy, Shandong Agricultural University, Tai-an, Shandong 271018, PR China*

[b]*Agronomy Department, Purdue University, 915 W State Street, West Lafayette, IN 47907, USA*

[†]These authors contributed equally to this work.

[*]Corresponding author. Tel.: +86 538 824 1485; Fax: +86 538 824 1485.

E-mail addresses: jwzhang@sdau.edu.cn (J. Zhang).

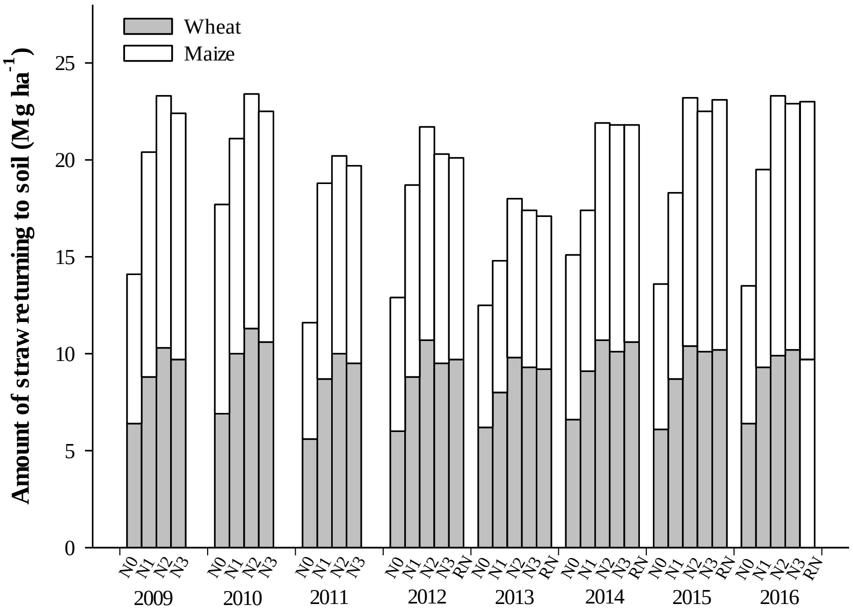

Supplement Fig. 1 The amount of straw produced and returned to the soil over the 2009-2016 study periods. Maize: summer maize residual straw. Wheat: winter wheat residual straw.

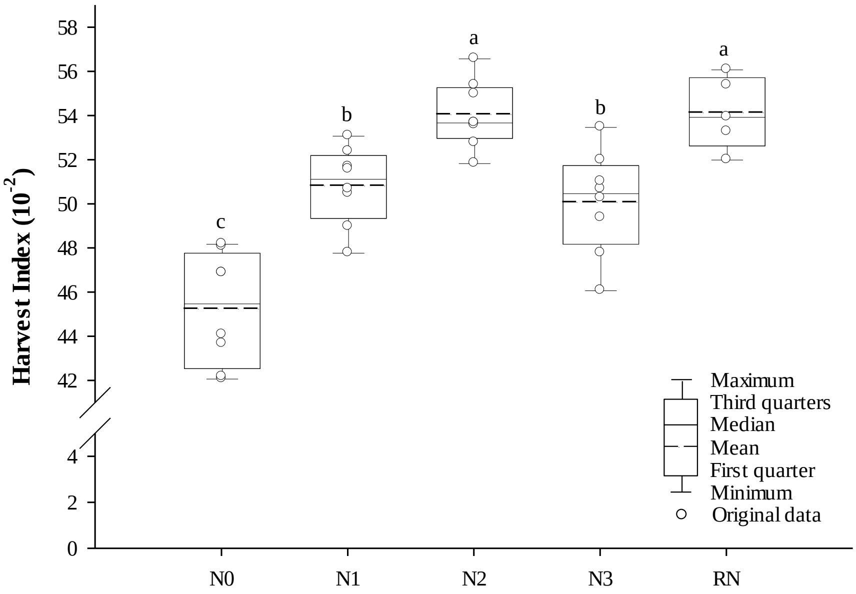

Supplement Fig. 2 Harvest index of summer maize as affected by nitrogen additions. Nitrogen addition in wheat and maize, respectively, are; 0 and 0 kg N ha^{-1} (N0), 168 and 129 N ha^{-1} (N1), 240 and 185 kg N ha^{-1} (N2 and RN2), and 300 and 300 kg N ha^{-1} (N3). Treatment means with different lower-case letters are significantly different at the $p \leq 0.05$ level.

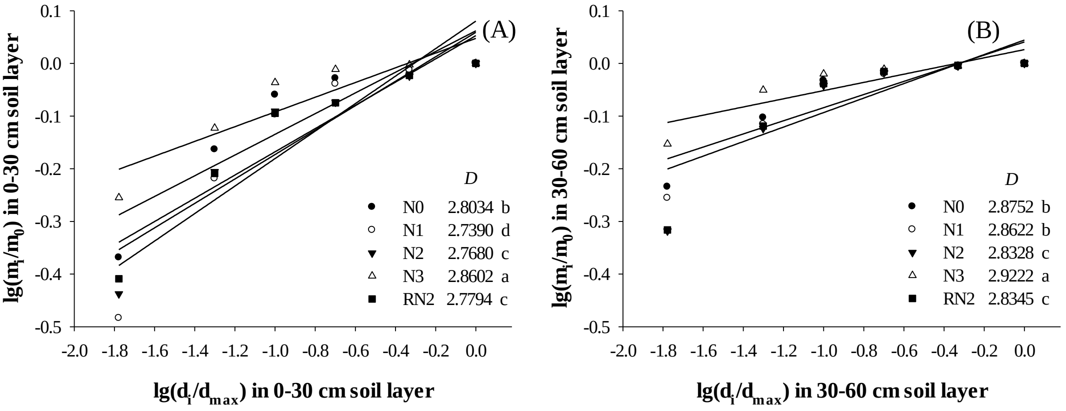

Supplement Fig. 3 Fractal dimension of water-stable aggregate in 0-30 (A) and 30-60 (B) cm soil layer. Nitrogen rates in wheat and maize, respectively, are; 0 and 0 kg N ha^{-1} (N0), 168 and 129 N ha^{-1} (N1), 240 and 185 kg N ha^{-1} (N2), and 300 and 300 kg N ha^{-1} (N3) in 2009-2016. Nitrogen rates under RN2 treatment in wheat and maize are 0 and 0 kg N ha^{-1} in 2009-2011, and 240 and 185 kg N ha^{-1} in 2012-2016. The different lower-case letters are significantly different at the $p \leq 0.05$ level. The water-stable aggregates include particles in diameter from 0.25 mm to 10 mm after wet-sieving. The parameters, m_i and d_i, represent cumulative weight and average diameter of specific water stable aggregates, respectively.

$D = 3 - \frac{\lg(m_i/m_0)}{\lg(\bar{d}_i/\bar{d}_{max})}$, which represent the fractal dimension of water-stable aggregates.

Supplement Table 1

Monthly precipitation and average temperature in 2009-2016.

Date (Year/Month)	Temperature (ºC)	Precipitation (mm)
2009/6	23.4	32.0
2009/7	25.7	68.0
2009/8	27.2	195.0
2009/9	20.1	216.0
2009/10	16.0	16.5
2009/11	6.9	12.9
2009/12	0.4	8.2
2010/1	-0.1	1.9
2010/2	2.6	7.9
2010/3	10.5	8.9
2010/4	16.6	17.4
2010/5	21.9	42.4
2010/6	25.3	45.0
2010/7	28.1	94.0
2010/8	26.4	226.0
2010/9	21.5	121.0
2010/10	15.4	17.7
2010/11	7.0	11.2
2010/12	0.8	3.0
2011/1	0.4	1.2
2011/2	2.8	8.6
2011/3	10.2	8.1
2011/4	16.2	19.8
2011/5	21.6	41.9
2011/6	25.9	39.0
2011/7	27.2	192.0
2011/8	25.4	165.0
2011/9	19.4	209.0
2011/10	16.5	24.3
2011/11	6.9	12.0
2011/12	1.0	4.4
2012/1	-0.2	1.6
2012/2	3.1	6.7
2012/3	10.3	11.5
2012/4	17.1	13.8
2012/5	20.4	42.8
2012/6	25.4	16.0
2012/7	27.3	210.5
2012/8	25.3	53.4
2012/9	20.2	61.6
2012/10	15.4	13.9
2012/11	6.5	17.8
2012/12	-0.9	28.4
2013/1	-1.6	5.6
2013/2	2.3	8.1

2013/3	9.3	12.1
2013/4	14.3	11.5
2013/5	21.9	57.7
2013/6	24.5	21.6
2013/7	27.4	349.8
2013/8	27.7	85.0
2013/9	21.3	49.4
2013/10	14.9	5.6
2013/11	6.9	15.2
2013/12	0.4	0.6
2014/1	1.5	0.0
2014/2	2.0	14.0
2014/3	10.6	0.6
2014/4	15.5	41.2
2014/5	20.5	53.8
2014/6	25.2	72.3
2014/7	27.9	261.7
2014/8	26.0	186.7
2014/9	22.1	94.8
2014/10	16.0	22.9
2014/11	7.5	14.2
2014/12	0.0	1.1
2015/1	1.0	2.0
2015/2	2.5	6.6
2015/3	9.5	17.9
2015/4	17.0	28.5
2015/5	21.0	66.9
2015/6	24.5	27.4
2015/7	28.0	185.4
2015/8	26.5	86.3
2015/9	22.0	358.4
2015/10	17.0	38.3
2015/11	6.0	14.6
2015/12	2.0	9.7
2016/1	-1.4	2.2
2016/2	3.7	10.9
2016/3	10.8	12.1
2016/4	18.2	6.7
2016/5	20.9	38.6
2016/6	25.9	35.6
2016/7	27.9	191.2
2016/8	26.9	201.0
2016/9	22.8	16.6
2016/10	16.3	26.2

Supplement Table 2

Ears per hectare, kernels per ear, and thousand-kernel weight (TKW) of summer maize as affected by N addition over an 8-year period. Nitrogen applied to wheat and maize, respectively; 0 and 0 kg N ha^{-1} (N0), 168 and 129 N ha^{-1} (N1), 240 and 185 kg N ha^{-1} (N2 and RN2), and 300 and 300 kg N ha^{-1} (N3).

Year	Treatment	Ears ha^{-1}	Kernels ear^{-1}	TKW, g
2009	N0	69287 a	439 c	327 b
	N1	69252 a	506 b	333 a
	N2	68388 a	513 a	337 a
	N3	69450 a	513 a	338 a
	RN2	-	-	-
2010	N0	63668 b	353 b	318 b
	N1	64540 b	469 a	323 b
	N2	67331 a	472 a	337 a
	N3	66033 a	461 a	337 a
	RN2	-	-	-
2011	N0	63753 a	418 c	288 b
	N1	65368 a	488 b	291 ab
	N2	64134 a	557 a	310 a
	N3	63893 a	573 a	304 a
	RN2	-	-	-
2012	N0	60682 b	481 b	258 c
	N1	65212 a	538 a	281 b
	N2	66371 a	541 a	297 a
	N3	66246 a	546 a	294 a
	RN2	66029 a	539 a	296 a
2013	N0	67556 c	429 c	261 c
	N1	70610 b	479 b	300 b
	N2	72726 a	493 a	307 a
	N3	72775 a	498 a	308 a
	RN2	72848 a	487 a	307 a
2014	N0	70333 c	369 c	285 b
	N1	72500 b	513 b	288 b
	N2	73489 a	543 a	303 a
	N3	73618 a	533 a	304 a
	RN2	73333 a	537 a	301 a
2015	N0	67198 c	411 c	318 c
	N1	70329 b	508 b	316 c
	N2	70418 b	541 a	341 a
	N3	70541 b	537 a	328 b
	RN2	70933 a	544 a	319 c
2016	N0	68279 c	505 b	310 c
	N1	72861 b	554 a	317 c
	N2	73463 ab	556 a	337 a
	N3	73860 a	556 a	327 b
	RN2	73631 a	554 a	336 a

Values followed by a different small letter in the same year are significantly different at $P \leq 0.05$ with Duncan’s multiple range test.

Supplement Table 3

Nitrogen addition effects on summer maize dry matter at tasseling (VT) and physiological maturity (R6) and the proportion of dry matter accumulated after VT (PDMA).

Year	Treatment	VT (g $plant^{-1}$)	R6 (g $plant^{-1}$)	PDMA(%)
2009	N0	96 c	235 c	59 b
	N1	122 a	310 b	61 b
	N2	110 b	331 a	67 a
	N3	101 c	328 a	69 a
	RN2	-	-	-
2010	N0	105 a	240 c	56 b
	N1	93 b	277 b	66 a
	N2	103 a	303 a	66 a
	N3	94 b	294 a	68 a
	RN2	-	-	-
2011	N0	87 b	182 c	52 b
	N1	91 ab	258 b	65 a
	N2	100 a	283 a	65 a
	N3	99 a	284 a	65 a
	RN2	-	-	-
2012	N0	82 b	191 c	57 c
	N1	97 a	263 b	63 b
	N2	99 a	288 a	66 a
	N3	100 a	284 a	65 a
	RN2	99 a	280 a	65 a
2013	N0	77 c	161 c	52 b
	N1	103 b	247 b	58 a
	N2	119 a	280 a	58 a
	N3	114 a	281 a	59 a
	RN2	108 b	273 a	60 a
2014	N0	86 d	211 c	57 c
	N1	97 c	252 b	61 b
	N2	107 b	311 a	66 a
	N3	114 a	313 a	63 b
	RN2	104 b	306 a	66 a
2015	N0	92 d	190 d	52 b
	N1	105 c	254 c	59 a
	N2	118 b	296 a	60 a
	N3	122 a	295 a	59 a
	RN2	121 a	293 a	59 a
2016	N0	91 c	190 d	52 b
	N1	110 b	254 c	56 ab
	N2	119 a	297 a	60 a
	N3	122 a	286 b	57 a
	RN2	123 a	304 a	59 a

PDMA = 100%×(R6－VT) / R6. Values followed by a different small letter in the same year are significantly different at $P \leq 0.05$ with Duncan's multiple range test.

Supplement Table 4

N content of summer maize straw and grain at physiological maturity and N harvest index (NHI) of summer maize as affected by N addition over 8 seasons. Nitrogen applied to wheat and maize, respectively, was; 0 and 0 kg N ha^{-1} (N0), 168 and 129 N ha^{-1} (N1), 240 and 185 kg N ha^{-1} (N2 and RN2), and 300 and 300 kg N ha^{-1} (N3).

Year	Treatment	Straw, kg N ha^{-1}	Grain, kg N ha^{-1}	NHI, %
2009	N0	89 d	120 c	58 a
	N1	122 c	155 b	56 b
	N2	136 b	165 a	55 b
	N3	145 a	161 a	52 c
	RN2	-	-	-
2010	N0	112 b	119 c	52 a
	N1	126 b	131 b	51 a
	N2	154 a	152 a	50 b
	N3	163 a	141 b	46 c
	RN2	-	-	-
2011	N0	80 d	101 b	55 a
	N1	90 c	108 b	54 a
	N2	117 b	131 a	53 b
	N3	139 a	117 b	46 c
	RN2	-	-	-
2012	N0	64 c	89 c	58 a
	N1	96 b	121 b	56 b
	N2	108 a	131 a	55 b
	N3	109 a	125 b	53 c
	RN2	102 a	131 a	56 b
2013	N0	76 d	97 d	56 a
	N1	102 c	121 c	54 b
	N2	134 b	154 a	53 b
	N3	142 a	131 b	48 c
	RN2	125 b	152 a	55 b
2014	N0	60 d	92 d	60 a
	N1	89 c	122 c	58 b
	N2	126 b	155 a	55 c
	N3	147 a	147 b	50 d
	RN2	129 b	153 a	54 c
2015	N0	60 d	94 c	61 a
	N1	93 c	120 b	56 b
	N2	134 b	147 a	52 c
	N3	155 a	146 a	49 d
	RN2	137 b	147 a	52 c
2016	N0	61 d	87 c	59 a
	N1	91 c	129 b	59 a
	N2	143 b	147 a	51 b
	N3	166 a	141 a	46 c
	RN2	139 b	145 a	51 b

NHI = 100%×grain N/(grain N + straw N); Values followed by a different small letter in the same year are significantly different at $P \leq 0.05$ with Duncan's multiple range test.

Supplement Table 5

Effects of N additions on N contribution proportion (NCP), N translocation efficiency (NTE), and N assimilation after anthesis (NA).

Year	Treatment	NCP (%)	NTE (%)	NA (kg ha^{-1})
2009	N0	50.13 b	40.41 a	59.38 d
	N1	51.48 b	39.52 a	75.36 b
	N2	58.66 a	41.76 a	68.43 c
	N3	42.98 c	32.11 b	91.40 a
	RN2	-	-	-
2010	N0	50.11 c	34.88 c	59.55 c
	N1	52.41 b	35.32 b	62.69 b
	N2	58.72 a	36.72 a	62.51 b
	N3	42.37 d	26.77 d	80.69 a
	RN2	-	-	-
2011	N0	49.89 c	38.17 b	50.14 c
	N1	52.49 b	38.47 b	51.03 c
	N2	59.67 a	40.38 a	53.18 b
	N3	42.85 d	26.60 c	66.72 a
	RN2	-	-	-
2012	N0	49.24 c	40.50 b	45.22 d
	N1	51.55 b	39.23 b	58.35 b
	N2	60.15 a	42.51 a	52.55 c
	N3	41.66 d	32.15 c	72.45 a
	RN2	58.33 a	42.72 a	54.54 c
2013	N0	48.57 c	38.14 b	49.64 d
	N1	52.68 b	38.10 b	56.92 c
	N2	59.89 a	40.65 a	61.55 b
	N3	41.57 d	27.84 c	76.63 a
	RN2	57.61 a	41.25 a	64.12 b
2014	N0	48.33 c	41.98 a	47.05 d
	N1	52.73 b	41.73 a	57.25 c
	N2	60.14 a	42.60 a	61.55 b
	N3	41.28 d	29.03 b	85.88 a
	RN2	59.37 a	41.42 a	62.27 b
2015	N0	50.98 c	41.85 b	45.59 c
	N1	55.61 b	41.25 b	52.82 b
	N2	64.33 a	45.76 a	52.08 b
	N3	48.29 c	30.37 c	75.49 a
	RN2	63.19 a	45.83 a	54.11 b
2016	N0	48.98 c	41.30 b	46.28 d
	N1	53.33 b	40.09 b	50.52 c
	N2	62.10 a	45.82 a	50.79 c
	N3	47.74 c	31.19 c	78.90 a
	RN2	62.74 a	46.21 a	53.58 b

NCP = N translocation from vegetative tissue from VT to R6 / grain N; NTE = N translocation from vegetative tissue from VT to R6 / N content at VT; NA = grain N minus N translocation from vegetative tissue from VT to R6. Values followed by a different small letter in

the same column are significantly different at P≤0.05 with Duncan's multiple range test.

Supplement Table 6

N content in 0-90 cm soil layer (SN), grain content in maize or wheat (GN-M or GN-W) and fertilizer N loss (FNL) in different N additions.

Indicator	Treatment	2009	2010	2011	2012	2013	2014	2015	2016
SN (kg ha^{-1})	N0	6469	6451	6368	6257	6182	6278	6061	5916
	N1	6524	6759	6944	7073	7232	7551	7558	7617
	N2	6575	6893	7160	7388	7614	7991	8057	8163
	N3	6599	7050	7462	7824	8153	8468	8464	8475
GN-M (kg ha^{-1})	N0	120	119	101	89	97	92	94	87
	N1	155	131	108	121	121	122	120	129
	N2	165	152	131	131	154	155	147	147
	N3	161	141	117	125	131	147	146	141
GN-W (kg ha^{-1})	N0	105	108	102	99	103	97	101	100
	N1	121	124	110	107	121	117	119	114
	N2	136	141	123	119	142	140	131	132
	N3	145	151	133	128	147	146	139	140
Contribution to GN (kg ha^{-1})	N1		28	15	40	42	50	44	56
	N2		66	51	62	96	106	83	92
	N3		65	47	65	78	104	90	94
Contribution to SN (kg ha^{-1})	N1		253	268	240	234	223	224	204
	N2		336	350	339	301	281	283	251
	N3		469	495	473	404	219	213	156
FNL (kg ha^{-1})	N1		16	14	17	21	24	29	37
	N2		24	25	25	29	39	60	83
	N3		66	58	62	118	277	297	350

Contribution to GN is the annual grain N content of N additions minus that of N0. Contribution to SN is the interannual changes in SN of N additions minus that of N0. FNL is the annual N addition minus contribution to GN and contribution to SN. Results is the average of 9 repeats.

Supplement Table 7

Effects of N additions on total and available nutrients in 0-30 cm soil layer.

Year	Treatment	Total N	Total P	Total K	Available P	Available K
		g kg^{-1}	g kg^{-1}	g kg^{-1}	mg kg^{-1}	mg kg^{-1}
2009	N0	0.83 a	0.84 a	18 a	20 a	126 a
	N1	0.84 a	0.86 a	18 a	20 a	125 a
	N2	0.82 a	0.85 a	19 a	20 a	126 a
	N3	0.83 a	0.85 a	19 a	19 a	126 a
	RN2	-	-	-	-	-
2010	N0	0.85 a	0.86 a	20 a	20 a	130 a
	N1	0.86 a	0.85 a	20 a	20 a	130 a
	N2	0.86 a	0.88 a	19 b	20 a	126 b
	N3	0.88 a	0.87 a	19 b	20 a	126 b
	RN2	-	-	-	-	-
2011	N0	0.91 b	0.85 a	21 a	20 a	137 a
	N1	0.92 b	0.88 a	21 a	20 a	136 a
	N2	0.94 a	0.87 a	18 b	20 a	125 b
	N3	0.95 a	0.86 a	18 b	20 a	124 b
	RN2	-	-	-	-	-
2012	N0	0.95 b	0.90 a	21 a	20 a	141 a
	N1	0.97 ab	0.93 a	20 a	20 a	139 a
	N2	0.96 b	0.91 a	18 b	20 a	125 b
	N3	1.00 a	0.91 a	18 b	20 a	125 b
	RN2	0.95 b	0.92 a	21 a	20 a	139 a
2013	N0	1.01 b	0.93 a	21 a	20 a	140 a
	N1	1.03 b	0.92 a	20 a	21 a	140 a
	N2	1.03 b	0.94 a	18 b	21 a	125 b
	N3	1.10 a	0.92 a	18 b	20 a	125 b
	RN2	1.03 b	0.94 a	18 b	20 a	125 b
2014	N0	1.03 b	0.95 a	21 a	21 a	141 a
	N1	1.06 b	0.95 a	20 a	21 a	140 a
	N2	1.08 b	0.97 a	18 b	21 a	124 b
	N3	1.14 a	0.94 a	18 b	21 a	125 b
	RN2	1.09 b	0.96 a	18 b	21 a	125 b
2015	N0	1.05 c	0.94 ab	21 a	21 a	140 a
	N1	1.04 c	0.91 b	21 a	21 a	140 a
	N2	1.09 b	0.99 a	18 c	20 a	122 c
	N3	1.18 a	0.98 a	19 b	21 a	130 b
	RN2	1.10 b	0.94 ab	19 b	21 a	125 c
2016	N0	1.01 c	0.89 b	21 a	21 a	135 a
	N1	1.10 b	0.87 b	21 a	21 a	138 a
	N2	1.09 b	0.96 a	19 c	20 a	121 b
	N3	1.16 a	0.96 a	20 b	22 a	134 a
	RN2	1.10 b	0.91 b	20 b	22 a	121 b

Values followed by a different small letter in the same year are significantly different at P≤0.05 with Duncan's multiple range test.

NS: Not significant, P>0.05. *, **: Significant at the 0.05 and 0.01 probability level, respectively.

Supplement Table 8

Effects of N additions on urease activity and invertase activity determined in 0-30, 30-60, and 60-90 cm soil depths at V6, VT, and R6 maize growth stages.

Soil layer (cm)	Treatment	Urease activity (μg NH_3-N g^{-1} h^{-1})			Invertase activity (mg glucose g^{-1} hr^{-1})		
		V6	VT	R6	V6	VT	R6
0-30	N0	63 c	71 c	63 c	1.34 d	1.39 d	1.33 d
	N1	74 b	83 b	75 b	1.55 b	1.61 b	1.53 b
	N2	81 a	92 a	84 a	1.62 a	1.69 a	1.61 a
	N3	60 c	68 c	61 c	1.44 c	1.52 c	1.47 c
	RN2	78 a	89 a	81 a	1.66 a	1.69 a	1.63 a
30-60	N0	48 a	53 a	50 a	0.56 a	0.59 a	0.54 a
	N1	43 b	47 b	44 b	0.46 b	0.51 b	0.45 c
	N2	39 c	43 c	39 c	0.47 b	0.48 bc	0.47 b
	N3	38 c	42 c	41 c	0.46 b	0.48 bc	0.47 b
	RN2	43 b	48 b	46 b	0.47 b	0.47 c	0.47 b
60-90	N0	41 a	42 a	41 a	0.47 a	0.48 a	0.45 a
	N1	40 a	43 a	37 b	0.46 a	0.45 a	0.45 a
	N2	37 b	40 b	34 c	0.44 a	0.45 a	0.43 a
	N3	36 b	39 b	37 b	0.45 a	0.44 a	0.42 a
	RN2	35 b	39 b	36 b	0.44 a	0.45 a	0.44 a

Results are presented as means over 2015 and 2016. Values followed by a different small letter in the same soil layer are significantly different at P≤0.05 with Duncan's multiple range test.

Supplement Table 9

Summary of ANOVA for parameters of plant (Table 1a) and soil characteristic (Table 1b) as influenced by Treatment and Year.

Table 9a

Effect	Grain yield	Ears	Kernels	TKW	HI	PDMA	PFP_N	AE_N	NCP	NTE	NA
Treatment (T)	**	**	**	**	**	*	**	**	**	**	**
Year (Y)	**	*	NS	NS	*	NS	*	**	*	NS	*
T×Y	NS	*	NS	NS	NS	NS	NS	NS	NS	NS	NS

NS: Not significant, P>0.05. *, **: Significant at the 0.05 and 0.01 probability level, respectively. Ears: ears per hectare; Kernels: kernels per ear; TKW: thousand-kernel weight; HI: harvest index; PDMA: proportion of dry matter accumulated after tasseling; PFP_N: nitrogen partial factor productivity; AE_N: agronomic nitrogen efficiency; NCP: nitrogen contribution proportion; NTE: nitrogen translocation efficiency; NA: nitrogen assimilation after anthesis.

Table 9b

Effect	BD	WSA	Organic matter	Total N	Total P	Total K	Available P	Available K	NO_3-N	UA	IA
Treatment (T)	**	**	**	**	NS	NS	NS	*	**	**	**
Year (Y)	NS	NS	**	**	*	NS	**	*	**	NS	NS
T×Y	NS	NS	*	*	NS	NS	NS	*	NS	NS	NS

NS: Not significant, P>0.05. *, **: Significant at the 0.05 and 0.01 probability level, respectively. BD: bulk density; WSA: weight percentage of water-stable aggregate; NO_3-N: nitrate-N concentration; UA: urease activity; IA: invertase activity.
